# The C-terminal LCAR of host ANP32 proteins interacts with the influenza A virus nucleoprotein to promote the replication of the viral RNA genome

**DOI:** 10.1093/nar/gkac410

**Published:** 2022-05-27

**Authors:** Fangzheng Wang, Carol M Sheppard, Bhakti Mistry, Ecco Staller, Wendy S Barclay, Jonathan M Grimes, Ervin Fodor, Haitian Fan

**Affiliations:** Sir William Dunn School of Pathology, University of Oxford, Oxford, UK; Department of Infectious Disease, Faculty of Medicine, Imperial College, London, UK; Department of Infectious Disease, Faculty of Medicine, Imperial College, London, UK; Sir William Dunn School of Pathology, University of Oxford, Oxford, UK; Department of Infectious Disease, Faculty of Medicine, Imperial College, London, UK; Department of Infectious Disease, Faculty of Medicine, Imperial College, London, UK; Division of Structural Biology, Henry Wellcome Building for Genomic Medicine, University of Oxford, Oxford, UK; Diamond Light Source Ltd, Diamond House, Harwell Science and Innovation Campus, Didcot, UK; Sir William Dunn School of Pathology, University of Oxford, Oxford, UK; Sir William Dunn School of Pathology, University of Oxford, Oxford, UK

## Abstract

The segmented negative-sense RNA genome of influenza A virus is assembled into ribonucleoprotein complexes (RNP) with viral RNA-dependent RNA polymerase and nucleoprotein (NP). It is in the context of these RNPs that the polymerase transcribes and replicates viral RNA (vRNA). Host acidic nuclear phosphoprotein 32 (ANP32) family proteins play an essential role in vRNA replication by mediating the dimerization of the viral polymerase via their N-terminal leucine-rich repeat (LRR) domain. However, whether the C-terminal low-complexity acidic region (LCAR) plays a role in RNA synthesis remains unknown. Here, we report that the LCAR is required for viral genome replication during infection. Specifically, we show that the LCAR directly interacts with NP and this interaction is mutually exclusive with RNA. Furthermore, we show that the replication of a short vRNA-like template that can be replicated in the absence of NP is less sensitive to LCAR truncations compared with the replication of full-length vRNA segments which is NP-dependent. We propose a model in which the LCAR interacts with NP to promote NP recruitment to nascent RNA during influenza virus replication, ensuring the co-replicative assembly of RNA into RNPs.

## INTRODUCTION

Influenza A viruses belong to segmented negative strand RNA viruses (sNSVs) and represent a major threat to human and animal health. The influenza A virus genome is composed of negative-sense single-stranded viral RNA (vRNA) segments, which are assembled into separate viral ribonucleoprotein (vRNP) complexes with a heterotrimeric RNA-dependent RNA polymerase and multiple copies of viral nucleoprotein (NP) (1–3). The viral polymerase is responsible for transcription and replication of the vRNA in association with host factors (4). During transcription, the polymerase copies vRNA into capped and polyadenylated mRNAs in association with host RNA polymerase II (4). During replication, the polymerase first copies the vRNA to generate complementary RNA (cRNA), which is assembled with polymerase and NP into a complementary ribonucleoprotein (cRNP) complex and serves as a template for vRNA synthesis. Multiple polymerase molecules are required for replication: in addition to the polymerase resident in the vRNP and cRNP that acts as a replicase, a *trans*-activating polymerase is required that specifically promotes the cRNA to vRNA step by assisting template realignment (5–8). Furthermore, an encapsidating polymerase which captures the nascent RNA strand and initiates its assembly into progeny RNP during both steps of replication is required (9). It has been proposed that during replication elongation, NP molecules are recruited to the growing nascent strand of cRNA and vRNA to ensure their co-replicative assembly into RNPs so that no exposed cRNA and vRNA is generated that could be recognized by innate immune sensors. Non-segmented NSVs (nsNSVs) encode an acidic phosphoprotein (P) which bridges the polymerase (L) and nucleoprotein (N), and recruits N to nascent replication products (3,10,11). The P protein also acts as chaperone of N preventing its oligomerization to guarantee the supply of monomeric RNA-free N to the nascent RNA strand (12–15). However, sNSVs lack an intrinsic P protein and it remains unclear how the virus recruits NP during the elongation step of viral genome replication.

Acidic nuclear phosphoprotein 32 (ANP32) family proteins are known as important host factors of influenza viruses specifically supporting viral genome replication (16,17). These proteins are composed of an N-terminal leucine-rich repeat (LRR) domain, followed by a C-terminal low-complexity acidic region (LCAR). A 33-amino acid insertion in avian ANP32A between the LRR and LCAR domains was found to be critical for the activity of avian influenza virus polymerase (18). In a cryo-EM study, the LRR (amino acid residues 1–158) bridges an asymmetric dimer of influenza virus polymerase heterotrimers, which has been proposed to act as a replication platform for the viral genome (9). Although full-length human ANP32A (huANP32A) or chicken ANP32A (chANP32A) were used to form complexes with influenza C virus polymerase, the structure of the LCAR could not be fully resolved due to its flexibility. A region that extends from LRR of chANP32A locates in a groove formed by both polymerase molecules. Despite not being fully modelled, this region is estimated to include 20–30 amino acid residues, suggesting the N-terminal 180–190 residues of chANP32A are in contact with the polymerase dimer while the rest of the protein which exclusively belongs to the LCAR could be solution accessible. We hypothesize that the highly acidic flexible LCAR could act as a molecular whip recruiting basic NP molecules to nascent RNA, thus mimicking the role of the P protein during nsNSV genome replication.

In this paper, we investigate the role of the ANP32 LCAR in influenza A virus genome replication. We show that the ANP32 LCAR is required for RNA synthesis during virus infection and interacts directly with NP. Two previously identified RNA binding grooves of NP contribute to the interaction. We also find that the LCAR is important for polymerase function in a minigenome assay using a full-length neuraminidase (NA) genome segment as template RNA. However, the LCAR is dispensable for RNA production from a 47-nucleotide(nt)-long template which can be replicated in the absence of NP (19). We propose that influenza virus uses host protein ANP32 for NP recruitment to nascent RNA during the elongation stage of viral genome replication.

## MATERIALS AND METHODS

### Cells, viruses and plasmids

Human embryonic kidney (HEK) 293T cells (293T) and the previously described ANP32A/ANP32B double knockout human embryonic kidney 293T cells (293T-DKO) (20) were cultured in Dulbecco's modified Eagle's medium (DMEM, Gibco) supplemented with 10% fetal bovine serum (FBS, Gibco). Madin-Darby bovine kidney (MDBK) cells and Madin-Darby Canine Kidney (MDCK) cells were cultured in minimal essential medium (MEM, Gibco) supplemented with 10% FBS and 2 mM l-glutamine (Gibco). All cells were maintained at 37°C with 5% CO_2_. Influenza A/WSN/33 (H1N1) virus was generated using the pHW2000 eight-plasmid system (21). Plasmids pcDNA-PB2, pcDNA-PB1, pcDNA-PA, pcDNA-NP (22) expressing the RNP components of influenza A/WSN/33 virus, and pPOLI-NA (23), pPOLI-NP47 (19) expressing vRNA templates have been described previously. Plasmids expressing gluc1-tagged A/Victoria/3/75 (H3N2) NP and gluc2-tagged ANP32 proteins were generated using overlapping PCR as described previously (24). Plasmids expressing truncated versions of ANP32 proteins were created by site-directed mutagenesis of the previously described pCAGGS-huANP32A, pCAGGS-human ANP32B (pCAGGS-huANP32B), and pCAGGS-chANP32A plasmids (25). All constructs were confirmed by Sanger sequencing.

### Viral infections

293T-DKO cells in six-well plates were transiently transfected with 2.5 μg of plasmids expressing the indicated ANP32 proteins or their truncated versions. Twenty-four hours post-transfection, cells were infected with influenza A/WSN/33 virus at a multiplicity of infection (MOI) of 5 in DMEM containing 0.5% FBS. Eight hours post-infection, cells were harvested for the extraction of total RNA, and RNA levels were analysed by primer extension.

### RNA isolation and primer extension assays

Total RNA was extracted from 293T-DKO cells in six-well plate using 500 μl TRI reagent (Sigma) according to the manufacturer's instructions and 2 μg of RNA were subjected to primer extension analysis as previously described (26). Briefly, RNA was reverse transcribed using SuperScript III reverse transcriptase (Invitrogen) with ^32^P-labeled NA or NP segment-specific primers. A primer targeting cellular 5S rRNA was included as an internal control. Transcription products were resolved by 6% or 12% denaturing PAGE with 7 M urea in TBE buffer and detected by phosphorimaging on an FLA-5000 scanner (Fuji). ImageJ was used to quantify cDNAs and values were normalized to the cDNA derived from the 5S rRNA control. The values for the ‘vector’ control were subtracted from the sample values. Data were analysed using Prism 8 (GraphPad).

### Minigenome assays

Approximately 80% confluent monolayers of 293T-DKO cells in six-well plates were transfected with plasmids pcDNA-PB1 (1 μg), pcDNA-PB2 (1 μg), pcDNA-PA (0.5 μg), and pcDNA-NP (2 μg) together with pPOLI-NA (0.2 μg) and plasmids encoding the indicated ANP32 proteins (2.5 μg) using Lipofectamine 2000 (Invitrogen) according to the manufacturer's instructions. For the NP-independent replication assay pcDNA-NP was omitted and the pPOLI-NA plasmid expressing NA vRNA was replaced with pPOLI-NP47 expressing a 47-nt short vRNA-like template derived from the NP segment. Total RNA from transfected cells was isolated using TRI reagent at the indicated time points post transfection. The extracted RNA was analysed by primer extension assay as previously described (26).

### Immunoblotting

huANP32A, huANP32B, chANP32A and their truncated versions were probed with rabbit anti-huANP32A (ab155148, Abcam), rabbit anti-huANP32B (ab200836, Abcam), and rabbit anti-chANP32A (AV40203, Sigma) antibodies. Beta-actin was probed with a mouse anti-beta-actin antibody (sc-47778, SCBT). Goat anti-rabbit and anti-mouse antibodies conjugated to horseradish peroxidase (HRP) were used as secondary antibodies. Detection was carried out using Amersham ECL Western blotting detection reagents (GE Healthcare).

### Recombinant protein production

Wildtype and mutant NP from influenza A/NT/60/1968 (H3N2) virus were cloned into pGEX-6P-1 vector (GE Healthcare) with an N-terminal GST tag followed by a PreScission protease site for expression in *Escherichia coli*. Proteins were purified on Glutathione Sepharose (GE Healthcare). GST-tagged or untagged NP were released with 25 mM reduced glutathione or cleavage with PreScission protease overnight, respectively, and further purified on a Superdex 200 Increase 10/300 GL column (GE Healthcare) using 25 mM HEPES-NaOH, pH 7.5, 150 mM NaCl and 5% (v/v) glycerol. Full-length and truncated GST-tagged huANP32A, huANP32B and chANP32A were cloned into pGEX-6P-1 as described above for NP. After purification on Glutathione Sepharose, GST-tagged ANP32 proteins were eluted using 25 mM reduced glutathione. Proteins were buffer exchanged in 25 mM HEPES-NaOH, pH 7.5, 150 mM NaCl and 5% (v/v) glycerol to remove glutathione using Amicon concentrators (Millipore). An empty pGEX-6P-1 vector was used to produce a GST tag as negative control. Untagged chANP32A (1–220) was produced following the same protocol as described above for NP.

### GST pull-down assays

All pull-down assays were performed in 25 mM HEPES-NaOH, pH 7.5, 150 mM NaCl, 5% (v/v) glycerol at 4°C. Approximately 200 μg bait (GST tag alone or GST tagged ANP32 proteins) was incubated with 50 μl Glutathione Sepharose (GE Healthcare) for 2–3 h. The beads were then washed once with the same buffer as specified above before being loaded with 100 μg analyte (wildtype or mutant NP). If indicated, a 1.5× or 4.5× molar excess (over NP) of a 29-nt RNA (5′-AGUAGAAACAAGGCCGUAUAUGAACAGA-3′, Dharmacon) was added together with NP. The beads were then incubated for another 3–5 h and washed 3 times with the same buffer as above. GST tag was cleaved overnight with 50 μg PreScission protease in the presence of 1 mM DTT to release un-tagged bait from the beads. Protein samples were analysed by SDS-PAGE. To analyse binding of huANP32A and NP from cell lysates to purified GST-NP and GST-huANP32A, respectively, 293T cells in six-well plates were transfected with 3 μg of pCAGGS-huANP32A or pcDNA-NP. Forty-eight hours post transfection cells were lysed for 1 h at 37°C in 500 μl of cell lysis buffer (50 mM HEPES–NaOH, pH 8.0, 150 mM NaCl, 25% (v/v) glycerol, 0.5% NP-40, 1 mM β-mercaptoethanol, 2 mM MgCl_2_, 1 mM PMSF, 1× complete EDTA-free protease inhibitor cocktail tablet (Roche)) in the presence or absence of 250 U of Benzonase (Sigma). 400 μl of cell lysate was applied to the beads with bait bound as described above. The beads were then incubated for another 3–5 h and washed 3 times before eluting overnight with 25 mM reduced glutathione. Samples were analysed by western blotting.

### Analytical size exclusion chromatography

Analytical size exclusion chromatography (SEC) experiments were performed on a Superdex 200 Increase 10/300 GL column (GE Healthcare) in 25 mM HEPES-NaOH, pH 7.5, 150 mM NaCl, 5% (v/v) glycerol at 4°C. In the analytical SEC of NP (R416A) and chANP32A (1–220), either a mixture of purified NP (R416A) and chANP32A (1–220), or a complex of the two from a GST pull-down, was loaded on the column. The mixture of purified NP (R416A) and chANP32A (1–220) (1:2 molar ratio of NP:ANP32A) was incubated on ice for 3 h before injection onto the column.

### Split luciferase assay

The assay was performed as described previously (24) with minor modifications. Briefly, control 293T or 293T-DKO cells were seeded at ∼65% confluency in 48-well plates. Cells were co-transfected with 20 ng pCAGGS expression plasmids encoding A/Victoria/3/75 (H3N2) NP-luc1 and chANP32A-luc2, huANP32A-luc2, huANP32B-luc2 or huANP32A 1–149 and incubated for 24 h at 37°C. Samples substituting pCAGGS-NP-luc1 with pCAGGS-luc1 and pCAGGS-NP, pCAGGS-ANP32-luc2 substituted with pCAGGS-luc2 and pCAGGS-ANP32 were used as background controls. Cells were lysed in 60 μl Renilla lysis buffer (Promega) with or without 10 ng/μl of RNaseA gently shaking for 1 h at room temperature. Gaussia luciferase activity was assayed using 20 μl of cell lysate and 100 μl of the Renilla luciferase reagent (Promega). Injection of substrate and measurement of bioluminescence were carried out using the FLUOstar Omega plate reader (BMG Labtech). Normalized luminescence ratios were calculated by dividing the signal from the chosen interacting partners by the sum of the two controls as described (27).

## RESULTS

### The LCAR of ANP32 proteins is pivotal to viral RNA synthesis during influenza virus infection

**Figure 1.**
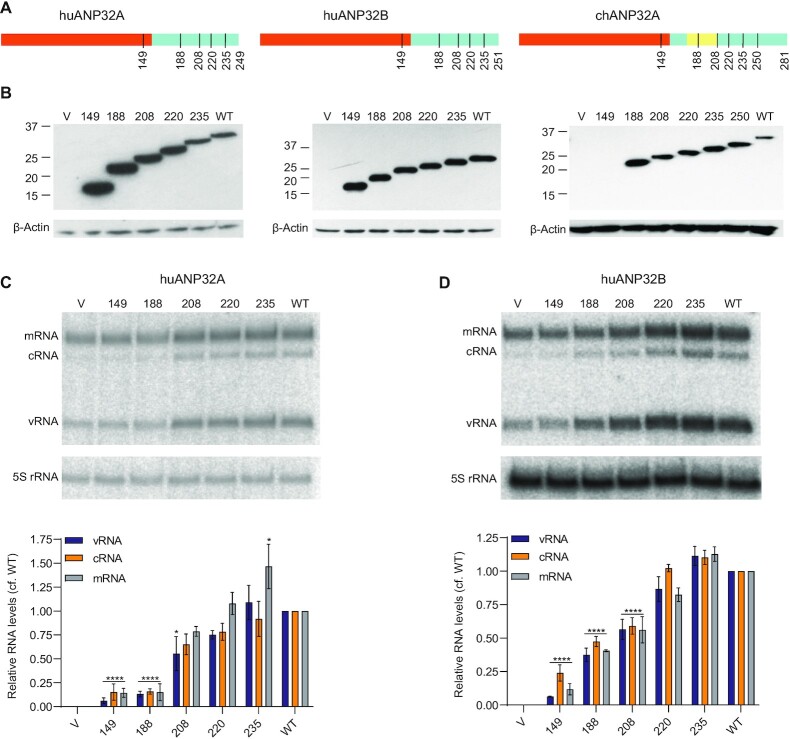
ANP32 LCAR is required for viral RNA accumulation during infection. (**A**) Schematic diagram of huANP32A, huANP32B, chANP32A and truncated mutants used in this study. Regions of LRR, LCAR, and the 33-amino-acid-insertion in chANP32A are coloured in red, blue, and yellow, respectively. The numbers under the schematics indicate the length of truncation mutants of ANP32 proteins. (**B**) Expression levels of wildtype (WT) or truncation mutants of ANP32 proteins in 293T-DKO cells. Note that the chANP32A-specific antibody does not recognize the 1–149 truncation mutant. Transfection of an empty vector (V) was used as a negative control. Molecular weight markers are indicated in kDa. (**C**, **D**) The effect of LCAR truncations on viral RNA synthesis during infection. 293T-DKO cells were transfected with plasmids to express the indicated huANP32A (C) or huANP32B (D) proteins. Twenty-four hours post-transfection cells were infected with influenza A/WSN/33 (H1N1) at an MOI of 5. Total RNA was isolated at 8 h post-infection and was analysed by primer extension assay. The quantitative results show the mean signal intensity (with the activity of vector subtracted) relative to that of the polymerase in the presence of wildtype ANP32 proteins from three independent experiments. Error bars represent the standard error of the mean (*n* = 3). Significance was assessed using Ordinary Two-way ANOVA and asterisks indicate a significant difference as follows: *P < 0.05; ****P < 0.0001.

We have previously reported the structure of a complex between the influenza virus polymerase and ANP32A (9). In the structure, the N-terminal LRR mediates dimerization of heterotrimeric polymerase molecules and we proposed this dimer provides a replication platform for the influenza virus RNA genome. However, most of the C-terminal LCAR remains unresolved in the structure and its role in influenza virus replication remains unclear. To address this, we constructed a series of truncated versions of ANP32 proteins (huANP32A, huANP32B and chANP32A) based on reported structural and functional studies (9,28) (Figure 1A).

To assess the effect of LCAR deletions on viral RNA synthesis in an infection scenario, we took advantage of the previously described double knockout human 293T cells that do not express huANP32A and huANP32B (293T-DKO) (20). All ANP32 constructs expressed equally well in 293T-DKO cells (Figure 1B). Truncated huANP32A or huANP32B proteins were pre-expressed in 293T-DKO cells prior to infection with influenza A/WSN/33 virus and RNA accumulation at 8 hours post-infection was analysed using primer extension (Figure 1C). Expression of full-length wildtype huANP32A (WT) resulted in a substantial increase of all three viral RNAs, including mRNA, cRNA and vRNA, compared to the control (vector only). Constructs retaining parts of the LCAR (1–208, 1–220 and 1–235) were also able to support viral RNA accumulation similar to wildtype (Figure 1C**)**. However, deletion of most of the LCAR (construct 1–188) or complete LCAR together with part of the LRR (construct 1–149) diminished RNA accumulation to basal levels (vector). Similar results were observed for huANP32B although construct 1–188 was able to support some activity while construct 1–208 showed some reduction, as compared to wildtype (Figure 1D). These results indicate that the LCAR of ANP32 proteins plays an important role in RNA synthesis during virus replication, which is consistent with recently published studies (24,28,29).

### The LCAR of ANP32 proteins interacts directly with NP

**Figure 2.**
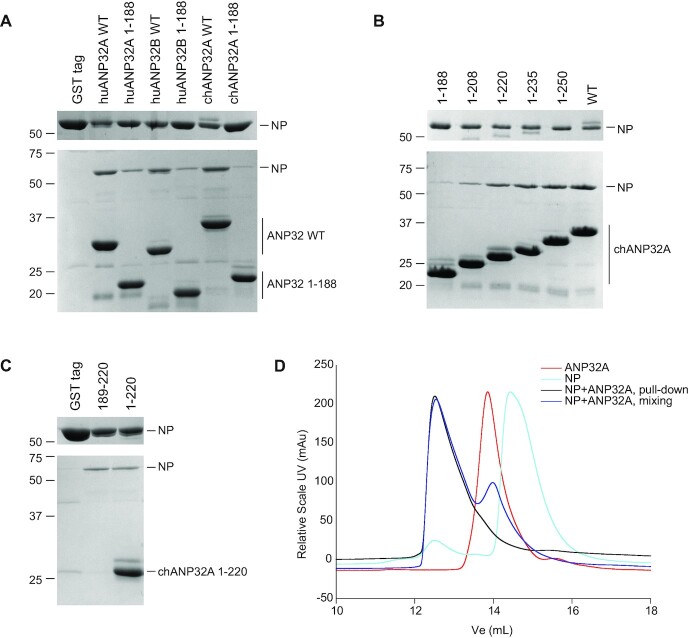
ANP32 LCAR interacts with influenza A virus NP. (A-C) GST pull-down assays using GST-tagged ANP32 proteins and NP. Wildtype (WT) or truncated (1–188) huANP32A, huANP32B and chANP32A (**A**), a series of chAPNP32A truncation mutants (**B**) and an LCAR peptide corresponding to amino acid residues 189–220 of chANP32A along with chANP32A 1–220 (**C**) with a cleavable N-terminal GST tag were immobilized on glutathione sepharose before the addition of NP with a R416A mutation. Bound proteins were released by treatment with PreScission protease. A purified GST tag alone was used as negative control (A, C). Unbound (upper gels) and released (lower gels) samples were analysed by SDS-PAGE and staining with Coomassie Brilliant Blue. Molecular weight markers are indicated in kDa. Note that the 189–220 LCAR peptide is too small to be captured on the gel. (**D**) Size exclusion chromatography of a complex of chANP32A 1–220 and NP R416A formed either using GST pull-down (pull-down) or mixing the two components (mixing).

We hypothesized that the highly acidic flexible LCAR could act as a molecular whip recruiting the basic NP to nascent RNA during influenza virus RNA genome replication. To address this, we performed assays using purified recombinant ANP32 proteins and NP. The NP we used is derived from influenza A/NT/60/1968 (H3N2) virus (30). Since purified wildtype influenza A virus NP forms oligomers (31,32), we introduced the point mutation R416A, which makes NP monomeric (33,34). In a GST pull-down assay, NP was found to interact with N-terminally GST-tagged, full-length huANP32A, huANP32B and chANP32A, while no interaction was observed between NP and the GST tag alone (Figure 2A). When using LCAR-truncated ANP32 proteins comprising amino acid residues 1–188, corresponding to the region that mediates influenza virus polymerase dimerization (9), much weaker interactions were observed, particularly with chANP32A (Figure 2A). These results indicate that the LCAR is the primary mediator of interaction between ANP32 proteins and NP.

To further investigate the ANP32-NP interaction, we used a series of LCAR-truncated chANP32A constructs (Figure 1A). The amount of bound NP increased as the length of ANP32 proteins increased from 1–188 to 1–281 (wildtype) (Figure 2B). Notably, there was a substantial increase in binding between the 1–188 and 1–220 constructs, suggesting that the region of 189–220 is particularly important for NP binding. To corroborate this finding, we expressed a peptide corresponding to region 189–220 of chANP32A and tested its ability to bind NP. We found that the peptide pulled down NP at the same level as chANP32A 1–220, indicating that the 189–220 region of the LCAR enables efficient binding to NP, while the LRR has much less contribution to this interaction (Figure 2C).

The interaction of chANP32A with NP was further tested by size exclusion chromatography. Complexes were prepared by either GST pull-down or pre-mixing individually purified proteins. Both samples resulted in an earlier elution peak on a Superdex 200 column, compared with either chANP32A or NP alone (Figure 2D). This indicates the complex formed by chANP32A and NP is stable in solution.

### RNA binding grooves of NP are involved in LCAR binding

**Figure 3.**
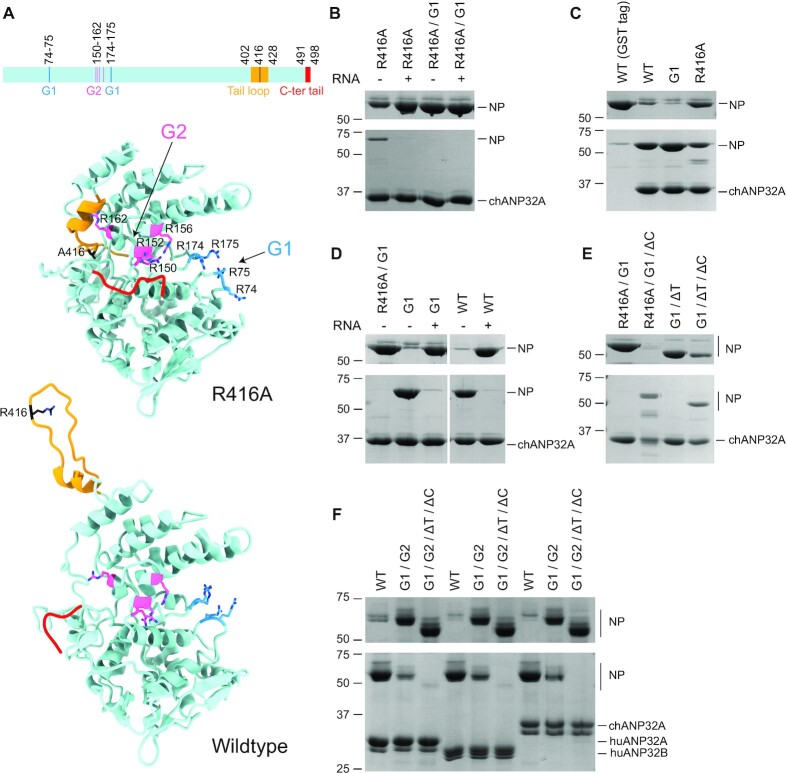
RNA binding grooves of NP are involved in the interaction with ANP32 proteins. (**A**) Schematic diagram and structural models of influenza A virus NP (R416A, PDB ID: 3ZDP, and wildtype, PDB ID: 2Q06) showing the G1 (blue) and G2 (pink) RNA binding grooves, tail loop (yellow), and C-terminal tail (red). Key amino acid residues in both the RNA binding grooves and tail loop are shown in stick mode in structural models. (**B–F**) GST pull-down assays using GST tagged chANP32A, huANP32A or huANP32B and wildtype or mutant NP in the absence or presence of 1.5× (+) molar excess of a 29-nt RNA (B, D). The following NP mutants were used: R416A, G1 (R74A/R75A/R174A/R175A), G2 (R150A/R152A/R156A/R162A), ΔT (amino acid residues 402–428 of the tail loop deleted), ΔC (amino acid residues 491–498 of the C-terminal tail deleted), and their combinations. ANP32 proteins with a cleavable N-terminal GST tag were immobilized on glutathione sepharose before the addition of wildtype or mutant NP. Bound proteins were released by treatment with PreScission protease. A purified GST tag alone was used as negative control (C). Unbound (upper gels) and released (lower gels) samples were analysed by SDS-PAGE and staining with Coomassie Brilliant Blue. Molecular weight markers are indicated in kDa.

To characterize the ANP32A binding site on NP, and to gain a better understanding of the ANP32A-NP complex, we made several NP mutants based on its structure (Figure 3A) and performed GST pull-down assays in the absence or presence of a 29-nt RNA. The presence of RNA severely reduced the interaction between a monomeric NP mutant (R416A) and chANP32A (Figure 3B). This suggests that RNA and the LCAR share the same binding interface on NP, and RNA possesses a stronger binding affinity to NP. To further analyse this, we used monomeric NP mutant (R416A) with four arginine to alanine mutations (R74A/R75A/R174A/R175A/R416A) in the G1 RNA binding groove (also known as the G1(4) mutant) (32,35). We found that this NP mutant, with or without the 29-nt RNA, was incapable of binding to chANP32A (Figure 3B). These data show that the G1 groove of NP contributes an important interface for the interaction with chANP32A.

Next we set out to test whether oligomeric NP could bind to chANP32A, using wildtype and a G1(4) mutant NP (R74A/R75A/R174A/R175A) in the assay. We found that wildtype NP bound to chANP32A but showed very little binding to the control GST tag (Figure 3C). Judging from band intensities, there appears to be more wildtype NP co-released with chANP32A post protease cleavage, compared with the R416A mutant. This could be due to multiple NP molecules binding to a single chANP32A resulting from its oligomerization. Surprisingly, we found that the G1(4) mutant showed chANP32A binding levels similar to that of the wildtype (Figure 3C). Presence of the 29-nt RNA strongly suppressed the interaction between chANP32A and the G1(4) mutant, as well as the wildtype NP (Figure 3D). These results suggest that multiple RNA binding sites of NP are likely to be involved in ANP32 protein binding.

In addition to the G1 RNA-binding groove, NP possesses a second RNA-binding groove referred to as G2 (32). Available structures of NP suggest that in the monomeric NP mutant (R416A), this site could be partially blocked by the tail loop (residues 402–428) and the C-terminal acidic tail (residues 491–498). The tail loop packs against a site next to the G2 groove and locates closely to R162; the C-terminal tail lies parallel to the G2 groove and locates closely to R150 and R152 (Figure 3A). In wildtype oligomeric NP, on the other hand, the tail loop reaches to the neighboring NP to form an inter-molecular salt bridge between R416 and E339 on the adjacent NP protomer; the C-terminal tail is either missing or partially modelled in a position opposite the RNA binding grooves (Figure 3A). Thus, key residues such as R150, R152 and R162 in the G2 RNA binding groove could be spatially blocked in the monomeric NP but remain exposed in the oligomeric state. These observations could explain why the monomeric and oligomeric forms of the G1(4) mutants show different affinities for chANP32A and led to the speculation that the G2 groove could contribute an additional chANP32A binding site.

To test this hypothesis, we generated several mutants of NP with either the tail loop (ΔT) or the C-terminal tail (ΔC) deleted. Removal of the C-terminal tail (residues 491–498) rescued chANP32A binding affinity of the monomeric G1(4) mutant, independent of whether the R416A point mutation or whole tail loop (402–428) deletion was used to prevent NP oligomerization (Figure 3E). These data suggest that in the monomeric NP, it is not the tail loop but the C-terminal tail that blocks the second NP-chANP32A interface, which is probably defined by the G2 groove. To address whether the G2 groove plays a role, we mutated all eight arginine residues to alanine in both G1 and G2 grooves. This NP mutant had substantially reduced affinity to chANP32A as well as huANP32A and huANP32B compared with wildtype NP (Figure 3F). The interaction was diminished in the monomeric version of such mutant with both the tail loop and C-terminal tail deleted. These data confirm the contribution of both G1 and G2 grooves to the ANP32 protein interaction.

### ANP32 proteins interact with NP in cells

**Figure 4.**
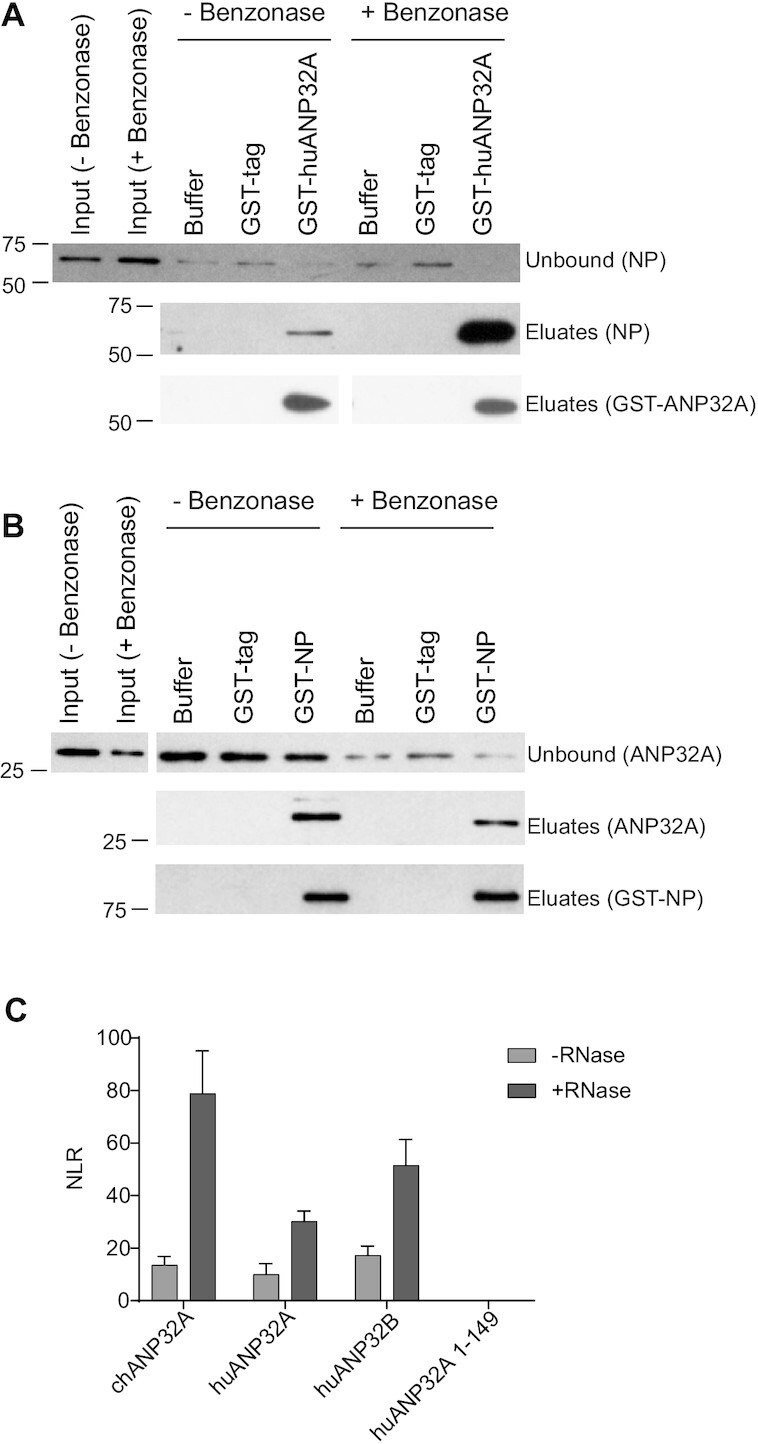
In-cell analysis of the interaction between ANP32 and NP. (**A**, **B**) Pull-down assays using bacterially expressed purified GST-tagged proteins and cell lysates from transfected cells. Input, unbound, and eluate samples were analysed by western blotting. Molecular weight markers are indicated in kDa. (**C**) Split luciferase interaction assays of ANP32 proteins and NP in 293T-DKO cells. Results shown are mean ± standard deviation from triplicate samples. NLR: normalized luminescence ratio.

To address whether ANP32 proteins and NP interact in cells, first we performed pull-down assays combining lysates of 293T cells expressing NP or huANP32A with purified recombinant GST-huANP32A or GST-NP expressed in bacteria. GST-huANP32A specifically pulled down NP and, in the reciprocal experiment, GST-NP specifically pulled down huANP32A from cell lysates (Figure 4A, B). GST-ANP32A pulled down significantly larger amounts of NP from cell lysates treated with the endonuclease Benzonase compared to lysates without Benzonase treatment while Benzonase treatment did not increase huANP32A binding to GST-NP in the reciprocal experiment. This result indicates that RNA bound to NP in cell lysates competes with huANP32A binding, in agreement with our data above that RNA interferes with the ANP32-NP interaction. Next, we performed a split luciferase assay with the N-terminal half of *Gaussia* luciferase (gluc1) fused to the C terminus of NP and the C-terminal half (gluc2) fused to the C terminus of huANP32A, huANP32B and chANP32A. We used huANP32A lacking the complete LCAR as negative control (huANP32A 1–149). We detected significant bioluminescence in lysates containing any of the three full-length ANP32 proteins and NP, compared with truncated ANP32A (Figure 4C). Luciferase activity further increased if RNaseA was included in the lysates, in agreement with the data above. Taken together, these data show that ANP32 proteins and NP interact in cells and RNA interferes with the interaction.

### The LCAR of ANP32 proteins is required for efficient replication of a full-length influenza genome segment but not a short vRNA-like template

**Figure 5.**
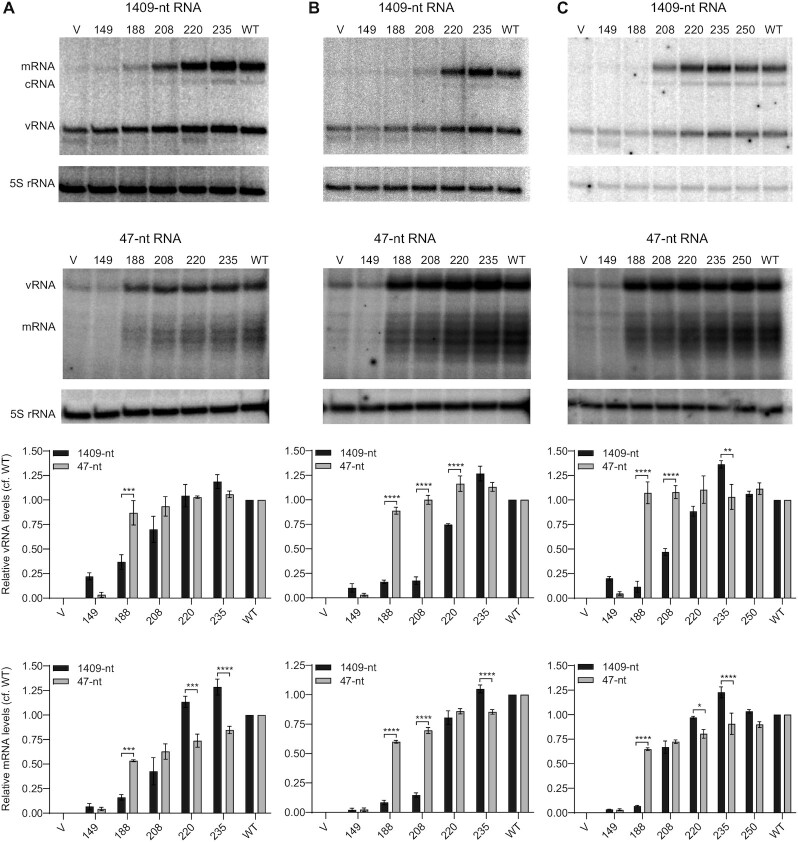
ANP32 LCAR is required for the replication of a full-length influenza genome segment but not a short vRNA-like template. (**A–C**) 293T-DKO cells were co-transfected with plasmids expressing the indicated wildtype (WT) or truncation mutant huANP32A (A), huANP32B (B) and chANP32A (C) proteins together with plasmids to express the PB1, PB2 and PA polymerase subunits, NP, and full-length NA vRNA (1409-nt) or a short vRNA-like template (47-nt) as indicated. Transfection of an empty vector (V) was used as a negative control. Total RNA was extracted at 24 hpt and the accumulation of vRNA, cRNA, and mRNA was analysed by primer extension assay. The quantitations show comparison of vRNA and mRNA accumulation for the full-length 1409-nt and short vRNA-like 47-nt templates observed in the presence of truncated ANP32 proteins relative to that observed in the presence of wildtype ANP32 proteins (with the values for the vector subtracted) from three independent experiments. Error bars represent the standard error of the mean (n = 3). Significance was assessed using Ordinary Two-way ANOVA and asterisks indicate a significant difference as follows: *P < 0.05; **P < 0.01 and ***P < 0.001, ****P < 0.0001.

Having established that the influenza virus NP interacts with the LCAR of ANP32 proteins, we next explored how LCAR deletions affect viral RNA replication that is dependent on NP. The requirement for NP during replication can be relieved when the full-length gene segment is replaced with a vRNA-like template that is shorter than 76-nts (19). Using a minigenome assay with either a full-length neuraminidase-encoding vRNA (1409-nt) or a 47-nt vRNA template in 293T-DKO cells, we tested the polymerase-supporting effect of truncated ANP32 proteins at 24 h post-transfection (Figure 5). Expression of huANP32A proteins retaining parts of the LCAR (1–220 and 1–235) resulted in the replication of both full-length and short vRNA templates at levels similar to that of the wildtype huANP32A (Figure 5A). The shortest version of huANP32A (1–149), which lacks part of the LRR domain that has been shown to associate with the polymerase dimer (9), supported the replication of neither full-length nor short vRNA template (Figure 5A). Importantly, huANP32A constructs with deletions of most of the LCAR (1–188 and 1–208) were able to support the replication of the short but not the full-length vRNA template. Similar results were observed with analogous truncation mutants of huANP32B and chANP32A (Figure 5B, C). These results suggest that NP-dependent replication of full-length templates is more sensitive to LCAR deletions than the replication of short vRNA-like templates that does not require NP.

**Figure 6.**
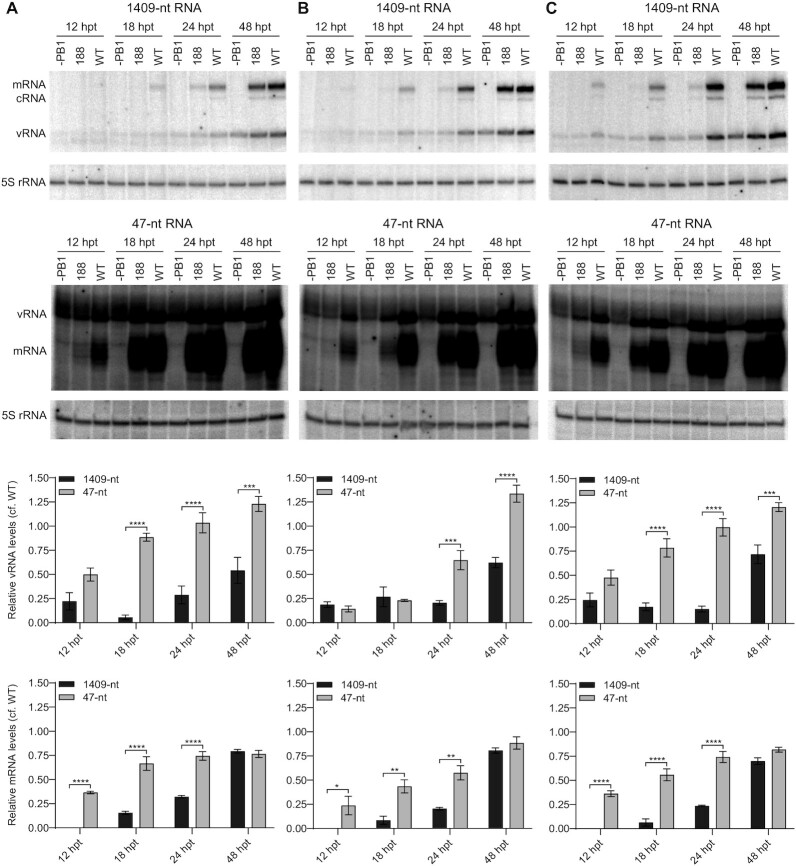
ANP32 LCAR truncation leads to delayed accumulation of viral RNAs during the replication of a full-length influenza genome segment compared to a short vRNA-like template. (**A–C**) 293T-DKO cells were co-transfected with plasmids expressing the indicated wildtype (WT) or 1–188 truncation mutant huANP32A (A), huANP32B (B), and chANP32A (C) proteins together with plasmids to express the PB1, PB2 and PA polymerase subunits, NP, and full-length NA vRNA (1409-nt) or a short vRNA-like template (47-nt) as indicated. The PB1 expression plasmid was omitted (–PB1) as a negative control. Total RNA was extracted at the indicated time points post transfection (hpt) and the accumulation of vRNA, cRNA, and mRNA was analysed by a primer extension assay. The quantitations show ratios of vRNA and mRNA accumulation in cells expressing wildtype and 1–188 ANP32 proteins for the full-length 1409-nt and short vRNA-like 47-nt templates from three independent experiments. Error bars represent the standard error of the mean (*n* = 3). significance was assessed using Ordinary Two-way ANOVA and asterisks indicate a significant difference as follows as follows: *P < 0.05; **P < 0.01 and ***P < 0.001, ****P < 0.0001.

To investigate this further we monitored the kinetics of viral RNA accumulation in 293T-DKO cells expressing truncated (1–188) or wildtype ANP32 proteins. Replication of the full-length template was substantially reduced in the presence of truncated huANP32A (1–188) compared to wildtype huANP32A at all time points tested. In contrast, replication of the short vRNA-like template only showed reduction at 12 h but by 24 h post transfection reached levels similar to those observed in the presence of wildtype huANP32A (Figure 6A). Changes in mRNA accumulation largely mimicked those in vRNA levels in agreement with vRNA serving as template for mRNA synthesis. We observed similar results with huANP32B (Figure 6B) and chANP32A (Figure 6C). Collectively, our results demonstrate that the LCAR of ANP32 proteins is more important for the replication of full-length vRNA segments compared to short vRNA-like templates that can be replicated in the absence of NP. These data are in agreement with our hypothesis that the LCAR is involved in recruiting NP to the nascent RNA during influenza virus RNA replication.

## DISCUSSION

Recently there has been considerable interest in the ANP32 family of proteins, essential host factors in influenza virus replication that work in concert with the viral RNA polymerase to mediate the replication of the viral RNA genome (18,20,24,25,28,29,36–45). Using structural studies, our group demonstrated that the ANP32 LRR is involved in mediating the dimerization of the influenza virus polymerase which we proposed is important for viral genome replication initiation (9). However, the role of the ANP32 LCAR, which is largely unresolved in the available structures, remains poorly understood. In this paper, we demonstrated the importance of the ANP32 LCAR in RNA synthesis during viral infection. Using pull-down and split luciferase assays, we showed that ANP32 proteins interact directly with NP via the LCAR. Mutagenesis of NP revealed that the G1 and G2 RNA binding grooves of NP contribute to the NP-ANP32 interface. Consequently, we found that RNA interferes with the ANP32-NP interaction and nuclease treatment resulted in increased association between ANP32 and NP in cell lysates. The presence of RNA in cell lysates could explain why this interaction was not detected in previous studies (36). We also showed that the replication of a full-length viral genome segment is dependent on the LCAR while the NP-independent replication of short vRNA-like templates (19,46) is less sensitive to LCAR deletions. Interestingly, we observed a reduction in viral RNA levels when truncated ANP32 is expressed compared to the wildtype for the short template at early time points post transfection (Figure 6). We speculate that this could be due to the ANP32 LCAR having functions beyond NP recruitment. For example, in the absence of structural information on the complete LCAR in the context of a polymerase-ANP32 complex we cannot exclude that the LCAR makes contacts with the polymerase that contribute to the formation of the replicase complex. Nevertheless, the overall higher levels of RNA present with the short template compared to the long template at all time points tested suggest the importance of the LCAR in NP recruitment.

Segmented NSVs do not encode an equivalent to the P protein expressed by non-segmented NSVs, therefore how free NP is recruited to the nascent RNA strand to form RNPs remains elusive. From data shown in this paper, we propose that influenza virus uses the LCAR of ANP32 proteins to substitute for the function of P protein in RNP assembly. The interactions between ANP32 proteins and NP are likely to be electrostatic due to the involvement of the highly acidic LCAR and basic grooves of NP. This is different from interactions between N and P proteins from non-segmented NSVs, which are mostly hydrophobic (47–52). In addition, P proteins are also proposed to act as chaperones to maintain N as monomeric before its association with nascent viral RNA. It is unclear whether ANP32 proteins could perform a similar function. Influenza viruses might use different strategies to maintain NP in monomeric form, for instance, using other host factors as molecular chaperones such as importins (53) or UAP56 (54), or alternatively, exploiting post-translational modifications such as reversible phosphorylation (34,55,56).

**Figure 7.**
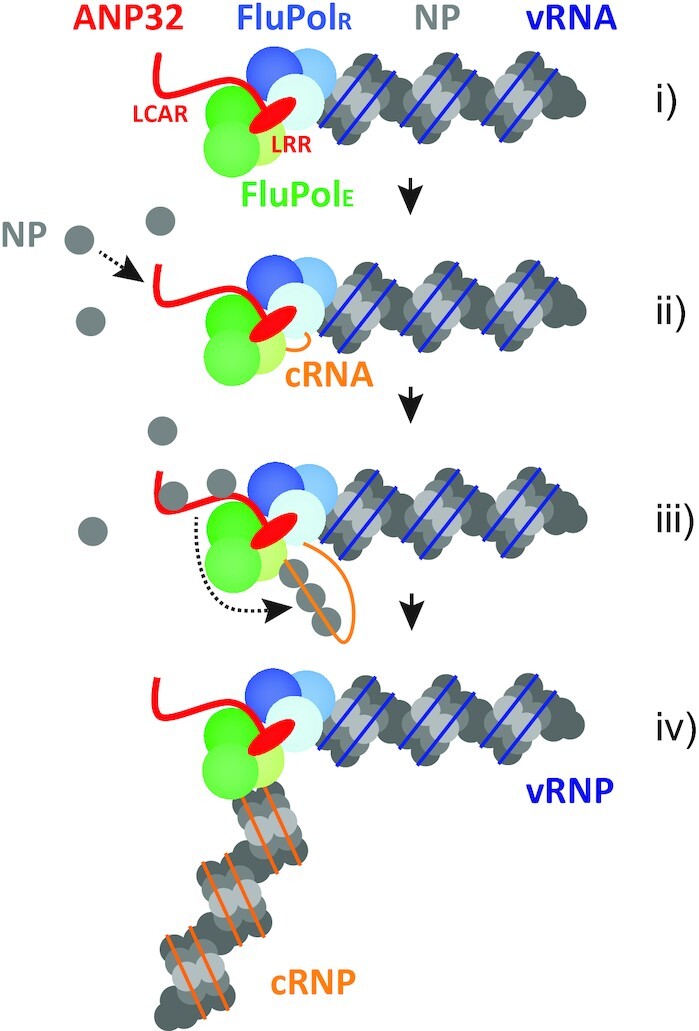
Model of influenza virus RNA genome replication. (i) ANP32 mediates the recruitment of a free influenza virus polymerase (encapsidating polymerase, FluPol_E_) to the polymerase resident in a vRNP (replicating polymerase, FluPol_R_) through its N-terminal LRR domain (red oval), leading to polymerase dimerization. (ii) FluPol_R_ initiates cRNA synthesis and the 5′ end of the nascent cRNA (orange) is captured by FluPol_E_. The flexible C-terminal LCAR of ANP32 (red line) recruits free NP molecules and increases local NP density. (iii) ANP32 LCAR facilitates the transfer of NP to nascent cRNA. NP dissociates from the LCAR and binds to cRNA due to its higher affinity to RNA. (iv) cRNA is assembled into a mature cRNP before being released from the template vRNP complex.

We propose a model in which the LCAR of ANP32 proteins is specifically involved in NP recruitment to nascent viral RNA. After polymerase dimerization mediated by the N-terminal LRR and replication initiation, the LCAR acts to capture monomeric RNA-free NP molecules to bring them spatially closer to the nascent RNA strand. As the nascent RNA strand extends, due to its higher affinity to RNA, NP dissociates from the LCAR of ANP32 proteins and binds to RNA through its RNA binding grooves, resulting in the assembly of progeny RNPs as this process repeats (Figure 7). In the absence of the LCAR domain, NP might still be able to bind to nascent RNA, albeit less efficiently (Figure 6). The length of the LCAR (100–130 amino acids) is sufficient to accommodate multiple NP monomers, suggesting that it could also serve to increase local NP density and thus enhance the efficiency of NP recruitment to viral RNA. To understand how the LCAR mediates recruitment of NP to nascent RNA it will be necessary to obtain structures of complexes of polymerase bound to ANP32A, nascent RNA and NP. The NPs of influenza A viruses share high sequence similarity at the putative ANP32 protein interaction interface (30), suggesting a conserved mechanism of influenza virus genome replication elongation across influenza A virus subtypes. As both cRNA and vRNA are assembled with NP into cRNPs and vRNPs, respectively, we propose that LCAR-dependent NP recruitment occurs during both vRNP and cRNP production. Indeed, a recent study reported the involvement of ANP32 family proteins in both influenza virus vRNA and cRNA synthesis (29). In conclusion, our paper reveals an important additional role of the ANP32 family of host proteins in influenza virus genome replication.

## DATA AVAILABILITY

Source data as well as plasmids are available upon request.
